# Supplementary material for: The Reticulon-4 3-bp Deletion/Insertion Polymorphism Is Associated with Structural mRNA Changes and the Risk of Breast Cancer: A Population-Based Case–Control Study with Bioinformatics Analysis
Source: Life (Basel). 2023 Jul 12;13(7):1549. doi: 10.3390/life13071549 (PMC10381770; doi:10.3390/life13071549)

Supplementary Figure S1. Sequencing results. Exemplar DNA sequencing result of RTN4 3-base-pair CAA rs34917480 polymorphism (A) insertion and (B) deletion allele

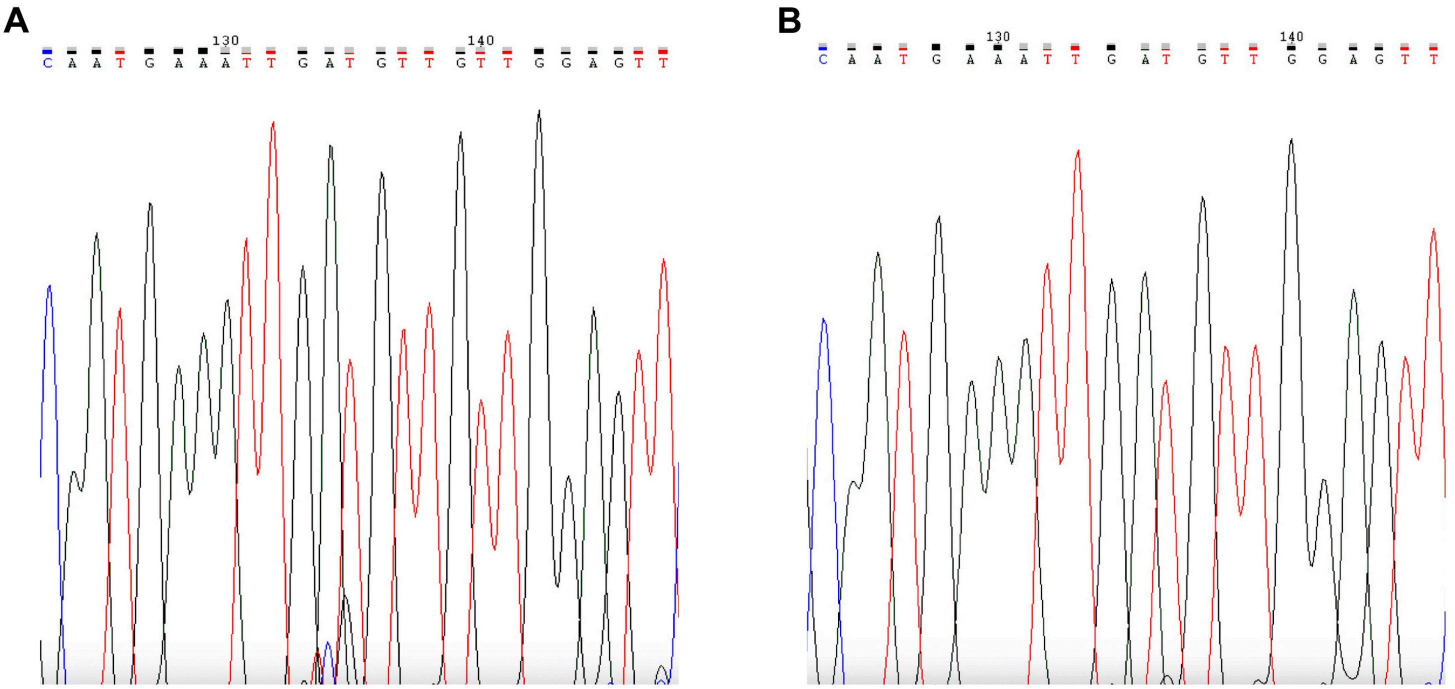

Supplement: Supplementary file 1 [file life-13-01549-s001.zip › supplementary figure S1.pdf]
